# Computational drug repositioning using similarity constrained weight regularization matrix factorization: A case of COVID‐19

**DOI:** 10.1111/jcmm.17412

**Published:** 2022-05-29

**Authors:** Junlin Xu, Yajie Meng, Lihong Peng, Lijun Cai, Xianfang Tang, Yuebin Liang, Geng Tian, Jialiang Yang

**Affiliations:** ^1^ College of Computer Science and Electronic Engineering Hunan University Changsha China; ^2^ School of Computer Science Hunan University of Technology Zhuzhou China; ^3^ Geneis Beijing Co., Ltd. Beijing China

**Keywords:** association prediction, drug‐target, drug–virus association, matrix factorization, similarity constrained

## Abstract

Amid the COVID‐19 crisis, we put sizeable efforts to collect a high number of experimentally validated drug–virus association entries from literature by text mining and built a human drug–virus association database. To the best of our knowledge, it is the largest publicly available drug–virus database so far. Next, we develop a novel weight regularization matrix factorization approach, termed WRMF, for in silico drug repurposing by integrating three networks: the known drug–virus association network, the drug–drug chemical structure similarity network, and the virus–virus genomic sequencing similarity network. Specifically, WRMF adds a weight to each training sample for reducing the influence of negative samples (i.e. the drug–virus association is unassociated). A comparison on the curated drug–virus database shows that WRMF performs better than a few state‐of‐the‐art methods. In addition, we selected the other two different public datasets (i.e. Cdataset and HMDD V2.0) to assess WRMF's performance. The case study also demonstrated the accuracy and reliability of WRMF to infer potential drugs for the novel virus. In summary, we offer a useful tool including a novel drug–virus association database and a powerful method WRMF to repurpose potential drugs for new viruses.

## 1 INTRODUCTION

Coronavirus (CoV) is currently one of the important viruses that endanger human health. It transmits through the respiratory tract of mammals, and causes mild‐to‐severe respiratory infections. In the past two decades, two highly pathogenic coronaviruses, including Severe Acute Respiratory Syndrome coronavirus (SARS‐CoV) and Middle East Respiratory Syndrome coronavirus (MERS‐CoV) have caused global epidemics of excessive morbidity and mortality in human community.^1^, ^2^ For example: In 2002, SARS‐CoV occurred in Guangdong, China, and spread to Southeast Asia and the world. About 8098 people were infected worldwide, of which 774 people died, which caused direct or indirect global economic losses of US $ 3 to 100 billion.^3^, ^4^ According to data from the World Health Organization (WHO), in addition to SARS‐CoV, MERS‐CoV began in Saudi Arabia in 2012. As of November 2019, it caused 2494 infections, including 858 deaths.^5^ In December 2019, a third pathogenic human coronavirus (HCoV) was discovered in Wuhan, China, which is known as a new enveloped RNA betacoronavirus2 named SARS‐CoV‐2.^6^, ^7^ On February 11, 2020, the World Health Organization named the new coronavirus‐infected pneumonia “COVID‐19”. As of July 30, 2020, there were more than 16.85 million COVID‐19 infections worldwide and more than 660,000 deaths. However, scientists still cannot find a special drug that could deal with all variants of SARS‐CoV‐2. In addition, scientific research teams in several countries are developing vaccines for the prevention and treatment of COVID‐19, but the incidences of infection are still rising. Therefore, there is an urgent need to find novel treatment plans for COVID‐19.^8^, ^9^

The development of a new drug for a disease (e.g. COVID‐19) is a long and expensive process. Therefore, drug repositioning is an effective drug discovery strategy, which can greatly reduce the time and cost compared with de novo drug discovery.^10^, ^11^, ^12^ Drug repositioning has been successfully applied in diseases like cancers.^13^, ^14^ However, how to prioritize potential drugs for specific diseases is still a bottleneck for drug repositioning. Research teams in various countries are constantly striving to find existing drugs to treat COVID‐19, For example, Draghici S et al. analysed the changes in the gene expression, pathways and putative mechanisms induced by SARS‐CoV2 and found that methylprednisolone (MP) could improve outcomes in severe cases of COVID‐19.^15^ But there are few drugs effective for COVID‐19 so far.^16^ Therefore, there is an urgent need for novel computational methods to repurpose drugs for COVID‐19.

The computational drug repositioning method provides new testable hypotheses for repositioning old drugs, which can predict potential drug–target interactions to direct the experimental verification and improve the drug discovery efficiency. In recent years, many computational association prediction methods have been developed. For example, Iorio et al. proposed a transcriptional‐network based approach, which applied the network theory and utilized similarity in gene expression profiles following drug treatment for drug repositioning.^17^ Sirota et al. developed a systematic computational approach based on compendia of public gene expression data to predict novel therapeutic indications.^18^ Peyvandipour et al. proposed a systems biology approach by considering the different roles of genes and their dependencies at the system level.^19^ Saberian et al. designed a novel machine learning‐based drug repositioning algorithm based on the theory that the distances between disease and its associated FDA‐approved drugs are smaller than that of other disease‐drug pairs.^20^ Martínez et al. presented a new network‐based methodology (called DrugNet) by constructing a heterogeneous network including drugs, proteins, and diseases.^21^ Yang et al. proposed a bounded nuclear norm regularization (BNNR) method to complete the drug–disease matrix for the prediction of drug–disease associations.^22^ Luo et al. proposed a novel network‐based method, called MBiRW, which uses some comprehensive similarity measures and Bi‐Random walk (BiRW) algorithm.^23^ Zeng et al. integrated ten networks (i.e. one drug–disease, one drug‐side‐effect, one drug–target, and seven drug–drug networks) and proposed a deep‐learning based method (named deepDR), consisting of a multi‐modal deep auto‐encoder and a collective variational auto‐encoder.^24^ Li et al. developed a new neural induction matrix completion method of the graph convolutional network (termed NIMCGCN).^25^ NIMCGCN was first utilized to predict miRNA–disease associations and was proven to have great potential in drug repositioning.

The above‐mentioned computational prediction approaches are mainly classified as network‐based approaches and machine learning‐based approaches. As the most typical machine learning‐based method, matrix factorization represents drugs and diseases in a shared latent space and reconstructs the drug–disease association using their latent vectors. Recently, a few variants of matrix factorization have also been widely and successfully used in bioinformatics researches,^26^ such as prediction of drug–drug interaction.^27^, ^28^ predicting drug side effects,^29^ predicting drug–target interactions,^30^ identifying drug–disease associations,^31^, ^32^ anticancer drug response prediction in cell lines,^33^ potential miRNA‐disease association prediction,^34^, ^35^, ^36^ and imputing the dropout entries of a given single‐cell RNA‐sequencing expression matrix.^37^ However, drug repositioning against human coronavirus like COVID‐19 prediction with limited information is challenging and meaningful.

In this study, we developed a new weight regularization matrix factorization method (WRMF) for drug repositioning against COVID‐19 based on similarity constraints, which mainly includes the following four steps: (i) collect experimentally verified drug–virus associations from the literature, (ii) calculate the chemical structure similarity of drugs and the genome sequence similarity of viruses, (iii) build heterogeneous networks based on known drug–virus associations, drug–drug similarity and virus–virus similarity, and (iv) use the similarity constrained weight regularization matrix factorization method to predict drugs most likely to be effective on the virus. Via comprehensive evaluation on 5‐fold cross‐validation (CV), local leave‐one‐out‐cross‐validation (LOOCV), and two additional independent datasets, we found that WRMF achieved higher performance in comparison with several state‐of‐the‐art methods. To fully prove the reliability of WRMF, we further conducted a case study about MERS. The experimental results showed that six of the top ten WRMF‐predicted anti‐MERS drugs had been confirmed. We expect that WRMF‐predicted anti‐COVID‐19 drug candidates might have a therapeutic effect. In summary, WRMF provides a powerful model to predict new drug–virus associations for accelerating drug repurposing.

## 2 MATERIALS AND METHODS

**FIGURE 1:**
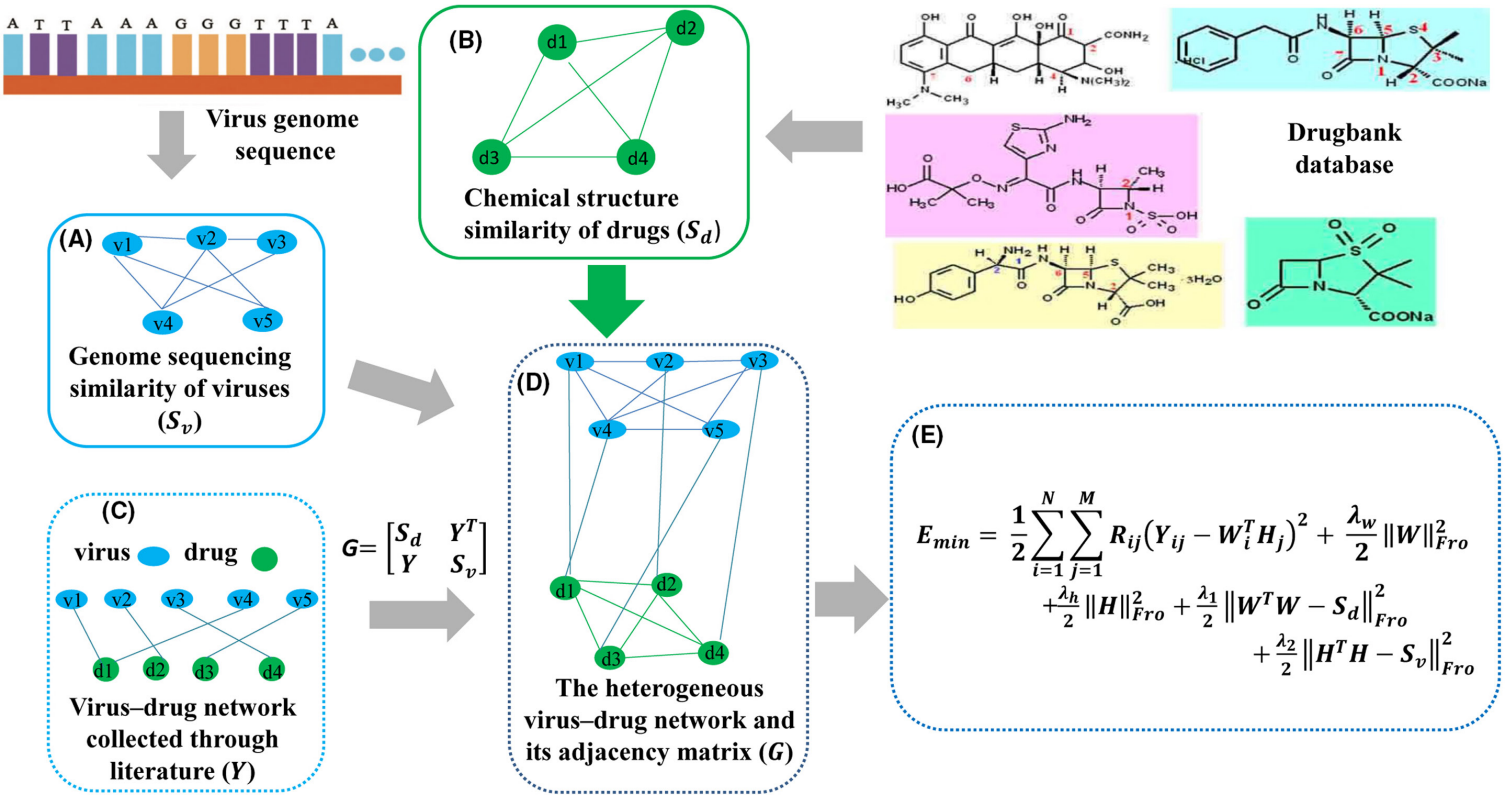
The workflow of WRMF

We give the main idea of WRMF in Figure 1, which mainly includes the following four steps: (i) collect data by searching literature to construct a data set; (ii) calculate (the similarity between viruses and the similarity between drugs; (iii) build a heterogeneous network based on existing data; and (iv) use the similarity constrained weight regularization matrix factorization method on heterogeneous networks to obtain potential viral therapeutics.

### 2.1 Human drug–virus association network

Since the databases of drug–virus associations are urgently demanded in biomedical researches amid the COVID‐19 crisis, Mongia et al. curated a comprehensive Drug Virus Association database, called DVA, which includes 38 viral species and 119 drugs.^38^ Meanwhile, we put sizeable efforts to collect a high number of experimentally validated drug–virus association entries from literature by text mining and built an experimentally supported human drug–virus association dataset consisting of 34 human infectious viruses, 218 therapeutic drugs, and 451 known human drug–virus associations (i.e. the drug is observed to have a known therapeutic role in the virus). Compared with DVA, the viruses we collected are mainly human‐infected coronaviruses and RNA viruses. In addition, we included 218 antiviral and broad‐spectrum drugs, which contains nearly 100 more drugs than DVA. As far as we know, our dataset is the largest in the sense that it contains the largest number of drugs and drug–virus associations.

We define the adjacency matrix of the drug–virus association network as the variable Y, that is, if the drug $d\left(i\right)$ is observed to have a therapeutic effect on the virus $v\left(j\right)$, the entity $Y\left(i,j\right)$is equal to 1; otherwise, it is 0. The two variables $n_{d}$ and $n_{v}$ represent the number of drugs and viruses, respectively. In this study, we integrate the drug–virus association network, drug–drug similarity network, virus–virus similarity network into a heterogeneous network. For the drug–drug similarity network, we measure the similarity of drug pairs by calculating the chemical structure similarity. For the virus–virus similarity network, we evaluate the similarity of virus pairs by calculating the gene sequences similarity. Therefore, the adjacency matrix of the drug–virus heterogeneous network can be defined as:

$$G=\left[\begin{array}{cc} S_{d} & Y^{T}\\ Y & S_{v} \end{array}\right]$$

The sub‐matrix Y represents the collected drug–virus association network, $Y^{T}$ is the transposition of Y, $S_{d}$, and $S_{v,}$ respectively, represent the adjacency matrix of drug–drug similarity network and virus–virus similarity network.

### 2.2 Chemical structure similarity of drugs

There are many algorithms for calculating drug similarity, among which classic algorithms usually include molecular similarity.^39^ In this article, we use the Tanimoto coefficient to express the similarity between drugs. The chemical structure information (SMILES format) was downloaded from the DrugBank database, and the MACCS fingerprint of each drug was calculated using Open Babel v2.3.1. If the MACCS fragment bit strings of two drug molecules are set with bits a and b, then c is set in the fingerprints of the two drugs, and the Tanimoto coefficient (T) of a drug pair is defined as:

(1) $$T=\frac{c}{a+b-c}$$

T is widely used in various drug development and relocation processes, and its value ranges from zero (no common bits) to one (all bits are the same).

### 2.3 Genome sequencing similarity of viruses

With the development of gene sequencing technology, our understanding of any virus often starts with its sequence. MAFFT is a multiple sequence alignment program for Unix‐like operating systems.^40^ It offers a range of multiple alignment methods, L‐INS‐i (accurate; recommended for <200 sequences), FFT‐NS‐2 (fast; recommended for >2000 sequences), etc. In the research of this paper, we use MAFFT to calculate the sequence similarity between viruses to express the similarity of viruses.

### 2.4 WRMF

The drug repositioning against Human Coronavirus Like COVID‐19 problem can be modelled as a recommendation system that recommends novel indications by filling out the unknown entries in the drug–virus association matrix, which is known as matrix completion. Matrix completion algorithms have been widely and successfully used in bioinformatics research, such as uncovering lncRNA–disease associations,^41^ predicting miRNA‐disease associations,^42^, ^43^, ^44^ identifying drug–disease associations,^45^, ^46^, ^47^ and selecting anti‐viral drugs for COVID‐19.^48^ In our study, there are 451 confirmed human drug–virus associations in the database we collected, which indicates that the known drug–virus association matrix is sparse. Based on the premise that similar drugs tend to treat similar viruses, the hidden factors that control the drug–virus associations are highly correlated, which results in an also highly correlated data matrix, and thus the number of underlying independent factors is much smaller than the existing number of drugs or viruses. In other words, the underlying latent factors determining drug–virus associations are highly correlated, and the drug–virus matrix to be completed is low‐rank. In fact, many studies used matrix completion methods for similar bioinformatics by constructing low‐rank matrix approximations consistent with known association matrix.^22^, ^41^, ^47^

Generally, when the matrix is of low rank, the matrix factorization minimization problem can be expressed as:

(2) $$E_{\min}=\frac{1}{2}\sum_{i=1}^{N}\sum_{j=1}^{M}{\left(Y_{\mathrm{ij}}-W_{i}^{T}H_{j}\right)}^{2}+\frac{\lambda_{w}}{2}{\left\|W\right\|}_{\mathrm{Fro}}^{2}+\frac{\lambda_{h}}{2}{\left\|H\right\|}_{\mathrm{Fro}}^{2}$$

where ${\left\|\cdot \right\|}_{F}$ represents the Frobenius norm, $\lambda_{w}$ and $\lambda_{h}$ are regularization parameters.

In the drug–virus association database, there are many unobserved entries, and we do not know negative samples (i.e. the drug–virus pair is unassociated). We define a problem with only positive feedback as a type of one‐class problem because there are only positive samples.^49^, ^50^ For one‐class problems, we proposed a novel weight regularization matrix factorization approach, which adds weight R to each training sample for reducing the influence of unknown samples. R represents the confidence of the drug's preference for the disease. In addition, the traditional matrix factorization does not take into account the similarity between viruses and the similarity between drugs. To solve the aforementioned problems, we propose a weight regularized matrix factorization model (WRMF), formalized as follows:

(3) $$R=\left\{\begin{array}{cc} 1+\alpha & Y_{\mathrm{ij}}=1\\ 1 & Y_{\mathrm{ij}}=0 \end{array}\right.$$

(4) $$E_{\min}=\frac{1}{2}\sum_{i=1}^{N}\sum_{j=1}^{M}R_{\mathrm{ij}}{\left(Y_{\mathrm{ij}}-W_{i}^{T}H_{j}\right)}^{2}+\frac{\lambda_{w}}{2}{\left\|W\right\|}_{\mathrm{Fro}}^{2}+\frac{\lambda_{h}}{2}{\left\|H\right\|}_{\mathrm{Fro}}^{2}+\frac{\lambda_{1}}{2}{\left\|W^{T}W-S_{d}\right\|}_{\mathrm{Fro}}^{2}+\frac{\lambda_{2}}{2}{\left\|H^{T}H-S_{v}\right\|}_{\mathrm{Fro}}^{2}$$

Among them, the hyperparameter α controls the contribution of positive samples to model training.$\;\lambda_{w}$,$\;\lambda_{h}$, $\lambda_{1,}$and $\lambda_{2}$ are the regularization parameters.

Since the WRMF model is a fitting of matrix Y, directly using SGD optimization will face the problems of overfitting and training efficiency.^22^ Therefore, we use the gradient descent algorithm to learn model parameters. The specific optimization steps are followed as:

(5) $${\;W}_{\mathrm{ik}}^{\mathrm{new}}=W_{\mathrm{ik}}\frac{{\left(R\cdot \left(HY^{T}\right)+2\lambda_{1}\left(W\left(S_{d}\right)\right)\right)}_{\mathrm{ik}}}{{\left(R\cdot \left(HH^{T}W\right)\right)}_{\mathrm{ik}}+{\left(\lambda_{w}W\right)}_{\mathrm{ik}}+{\left(2\lambda_{1}\left(WW^{T}W\right)\right)}_{\mathrm{ik}}}$$

(6) $$H_{\mathrm{jk}}^{\mathrm{new}}=H_{\mathrm{jk}}\frac{{\left(R\cdot \left(\mathrm{WY}\right)+2\lambda_{1}\left(H\left(S_{v}\right)\right)\right)}_{\mathrm{jk}}}{{\left(R\cdot \left(HH^{T}W\right)\right)}_{\mathrm{jk}}+{\left(\lambda_{h}H\right)}_{\mathrm{jk}}+{\left(2\lambda_{2}\left(HH^{T}H\right)\right)}_{\mathrm{jk}}}$$

According to formula (5) and formula (6), iteratively update W and H until the local minimum of the objective function. Finally, the predicted drug–virus association matrix is $Y^{*}=W^{T}H$. The ith column of $Y^{*}$indicates the association score between virus $v_{i}$ and drugs. The larger the score, the more relevant it is.

### 2.5 Performance evaluation of WRMF

To evaluate the performance of the algorithm, we used the 5‐fold CV and local LOOCV. In the 5‐fold CV experiment, all known drug–virus associations are randomly divided into five equal and disjoint parts. Then, leave a part as a test set in turn, and the remaining four parts are used as a training set to train the model. The process is repeated for five times until all samples are predicted once. In the local LOOCV experiment, for each virus $v_{i}$, we remove all the known associations of the virus $v_{i}$ and build prediction model using the remaining data. We then calculate the relevance score of each node in the test dataset, and rank these nodes according to their scores. The higher the grade of the positive sample, the better the performance is. If the score of the marked node is higher than a given threshold θ, it is regarded as a positive sample for successful identified. If the score of the unlabeled node is lower than θ, it is regarded as a negative sample correctly identified. By changing h, the true positive rate (TPR) and false positive rate (FPR) can be calculated to obtain the receiver operating characteristic (ROC) curve.^22^, ^51^

(7) $$\mathrm{TPR}=\frac{\mathrm{TP}}{\mathrm{TP}+\mathrm{FN}}$$

(8) $$\;\mathrm{FPR}=\frac{\mathrm{FP}}{\mathrm{TN}+\mathrm{FP}}$$

TP and TN are the numbers indicating that the positive and negative samples are judged correct, respectively. FP and FN indicate the number of positive and negative samples that were judged wrong.

## 3 RESULTS

### 3.1 Comparison with the state‐of‐the‐art methods

To evaluate the performance of our proposed WRMF, we compared WRMF with five state‐of‐the‐art association prediction methods listed below.

- IMC (Chen et al.),^42^ a novel inductive matrix completion method, which is designed by Chen et al. for predicting miRNA‐disease associations.
- CMF (Shen et al.),^52^ a collaborative matrix factorization method for identifying potential miRNA‐disease associations. CMF is widely used in recommendation systems and has great potential in drug repositioning.
- MBiRW (Luo et al.),^23^ a novel network‐based method, which uses comprehensive similarity measures and Bi‐Random walk (BiRW) algorithm to predict drug–disease associations.
- BNNR (Yang et al.),^22^ a bounded nuclear norm regularization method to complete the drug–disease matrix for the prediction of drug–disease association.
- NIMCGCN (Li et al.), ^25^ a deep learning approach, which is a new neural induction matrix completion method of the graph convolutional network. It was first used to predict miRNA–disease associations.

In our proposed WRMF algorithm, the hyperparameter α, which controls the contribution of positive samples to model training, ranges from 1 to 10 and is set as 5. Meanwhile, there are four main parameters needed to be determined, including $\lambda_{w}$, $\lambda_{h}$, $\lambda_{1},$ and $\lambda_{2}$. Based on the drug–virus association dataset we constructed, we performed cross‐validation on the training dataset to tune the parameters, which are increasing from 0.1 to 1 with a step of 0.1, and the ones with the best AUC were selected. WRMF achieves the best performance when $\lambda_{w}=\lambda_{h}=0.3$ and $\lambda_{1}=\lambda_{2}=0.1$ (see Figure S1). To ensure a fair comparison, the parameters in the compared approaches are set to the best values according obtained by using grid search (see Figure S2). Specifically, like WRMF, we chose the optimal parameters (IMC: $\lambda_{1}=\lambda_{2}=1$; CMF: $\lambda_{d}=\lambda_{m}=0.5,\lambda_{l}=0.1$) in the same way. In BNNR algorithm, there are two parameters (i.e. α and β) needed to be determined, and we obtained the optimal parameters (α=1, β=10) by determined from {0.1, 1, 10, 100}. In MBiRW algorithm, α is chosen from $\left\{0.1,0.2,\ldots ,1\right\}$ and the optimal value of α is 0.5. The parameters landr are set as 2. For the NIMCGCN algorithm, we chose the optimal parameters α=0.4 from $\left\{0.1,0.2,\ldots ,0.9\right\}$, l=3, and t=2.

### 3.2 Performance of WRMF on our constructed drug–virus dataset in 5‐fold CV

**FIGURE 2:**
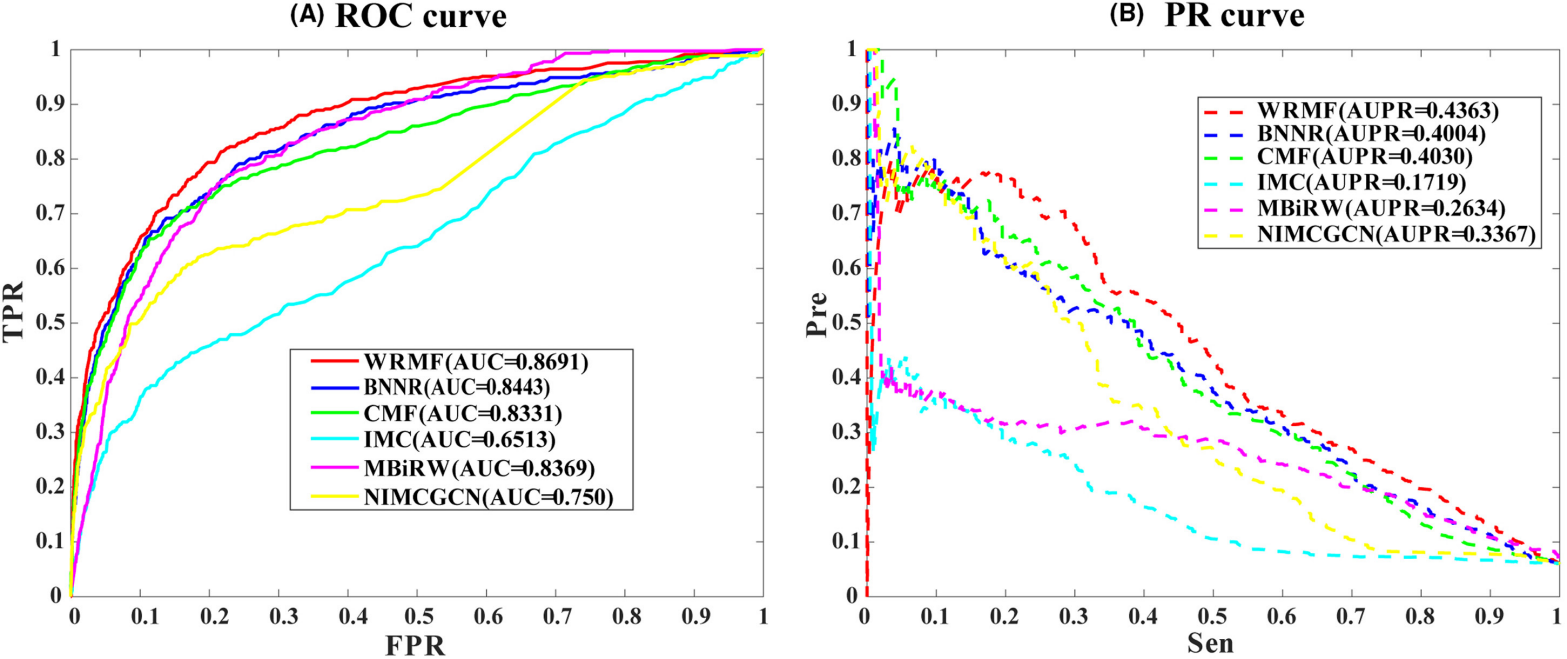
The performance of WRMF on our constructed drug–virus association dataset in comparison to the state‐of‐the‐art prediction methods. (A) ROC curve and AUCs value based on 5‐fold CV; (B) PR curve and AUPRs value based on 5‐fold CV

We applied WRMF, BNNR, CMF, IMC, MBiRW, and NIMCGCN into our constructed drug–virus association dataset, containing 451 unique associations between 34 viruses and 218 drugs. We plotted the receiver operating characteristic (ROC) curves and calculated the area under the ROC curve in the 5‐fold CV (see Figure 2A). As can be seen, AUCs of WRMF, BNNR, CMF, IMC, MBiRW, and NIMCGCN are 0.8691, 0.8443, 0.8331, 0.6513, 0.8369, and 0.750, respectively, indicating that WRMF performed the best in predicting drug–virus associations. Given the limited known drug–virus associations searched through the literature, we also used the precision‐recall (PR) curve and the area under the PR curve (AUPR) to comprehensively evaluate the performance (see Figure 2B). Generally, the PR curve shows similar changes to the ROC curve at different thresholds, and if the AUPR is close to 1, the prediction performance will be better. As shown in Figure 2B, the AUPRs of WRMF, BNNR, CMF, IMC, MBiRW, and NIMCGCN are 0.4363, 0.4004, 0.4030, 0.1719, 0.2634, and 0.3367. The AUPR obtained by WRMF is superior to those of the other methods, which again proves that WRMF performs best in drug repurposing.

### 3.3 Performance of WRMF on our constructed drug–virus dataset in local LOOCV

**FIGURE 3:**
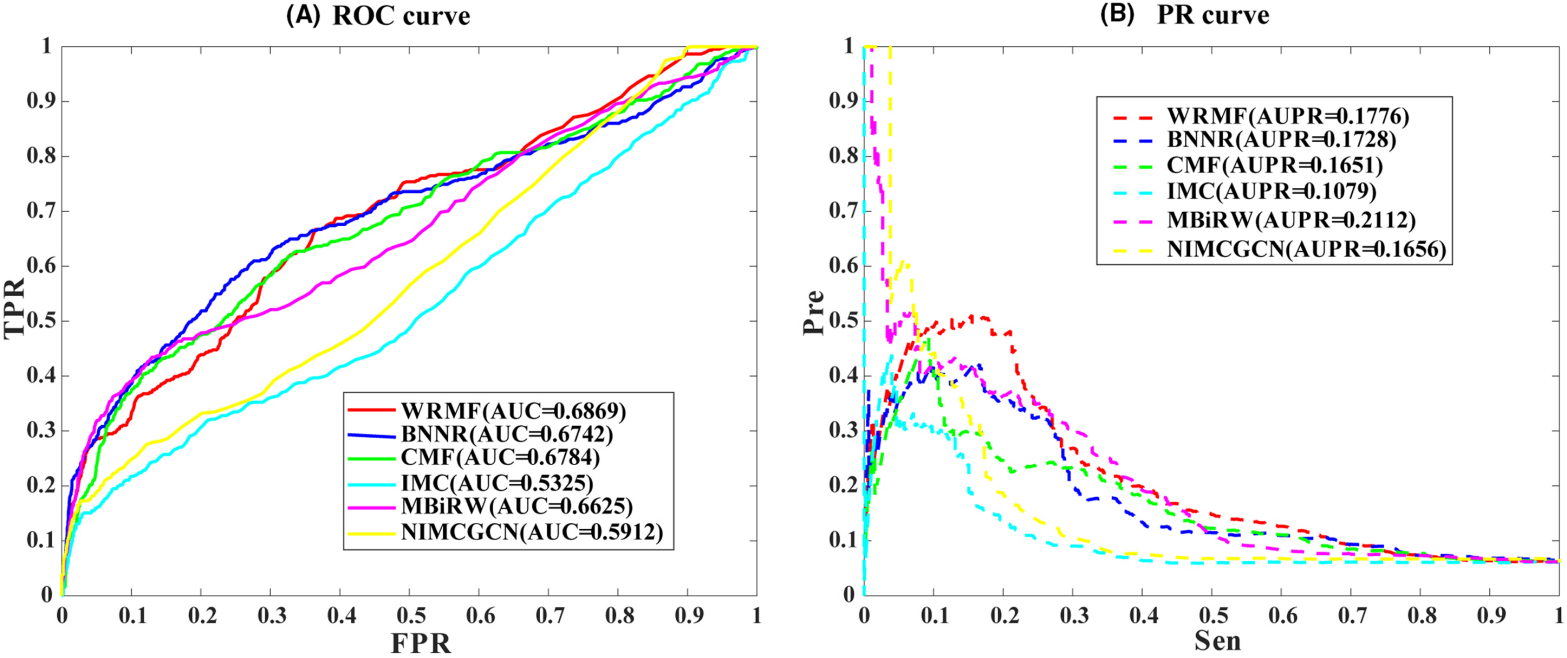
The prediction performance of WRMF on our constructed drug–virus association dataset by comparing with the other five published methods. (A) ROC curve and AUCs value based on local LOOCV. (B) PR curve and AUPRs value based on local LOOCV

Cross‐validation probably leads to over‐optimistic results because SARS‐CoV‐2 is a completely new virus. There was no connection between the drugs and COVID‐19. We further performed the local LOOCV to further evaluate the performance of WRMF. As can be seen in Figure 3A, the AUC of WRMF is the highest of all methods. In terms of AUPR (see Figure 3B), we find that WRMF achieves the second‐best performance (AUPR is 0.1776) in our constructed drug–virus dataset. The possible reason is that WRMF only uses drug chemical structure and virus genome sequence to calculate drug and virus similarity, while MBiRW considers the influence of known association information on the similarity measures and utilizes comprehensive similarity measures. In summary, WRMF has a good performance in predicting the potential therapeutic drugs of a new virus.

### 3.4 Performance of WRMF on two different types of datasets

In addition to the drug–virus association dataset collected by our study, we selected more challenging scenarios to assess the generalizable ability of WRMF. We compared WRMF with other three matrix factorization & completion methods (i.e. BNNR, CMF and IMC) on two different public datasets, which are the drug–disease association dataset (Cdatase)^23^ and the human microRNA disease database (HMDD V2.0),^53^ respectively.

**FIGURE 4:**
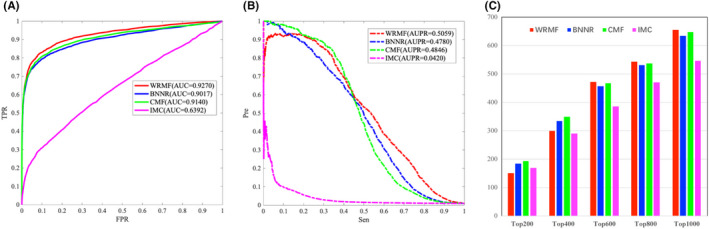
The performance of WRMF and other three matrix factorization & completion methods for predicting drug–disease association on Cdataset in 5‐fold CV. (A) ROC curves of the prediction results. (B) PR curves of the prediction results. (C) The number of confirmed drug–disease associations for various rank thresholds in top predictions of WRMF, BNNR, CMF, and IMC

Cdataset is generated by combining DNdatasets^21^ and the gold standard dataset,^54^ which contains 663 drugs collected in DrugBank, 409 diseases listed in the OMIM database, and 2352 known drug–disease associations. Figure 4 illustrates the performance comparison from the Cdataset. WRMF achieves a higher performance over the other comparison methods in terms of both AUC and AUPR. Specifically, WRMF obtains an AUC value of 0.9270 in 5‐fold CV, while BNNR, CMF, and IMC achieve AUCs of 0.9017, 0.9140, and 0.6392, respectively. The PR curves illustrate that WRMF obtains the highest AUPR of 0.5059, while those of BNNR, CMF, and IMC are 0.4780, 0.4846, and 0.0420, respectively. Moreover, WRMF identifies 827 associations in the top 1000 ranking, while BNNR, IMC, and CMF only predicted 815, 819, and 145 associations, respectively.

**FIGURE 5:**
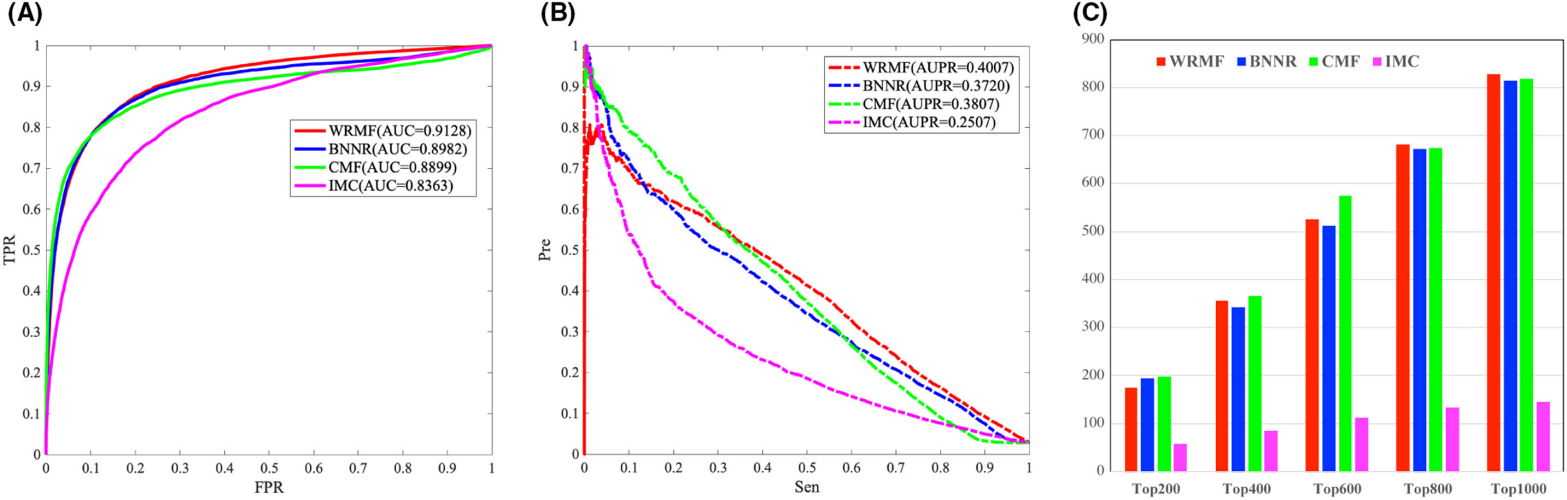
The performance of WRMF and other three matrix factorization & completion methods for predicting miRNA‐disease association on HMDD V2.0 in 5‐fold CV. (A) ROC curves of prediction results. (B) PR curves of predicting candidate miRNAs for diseases. (C) The number of confirmed miRNA‐disease associations for various rank thresholds in top predictions of WRMF, BNNR, CMF, and IMC

The effective identification of disease‐related miRNAs plays an important role in the discovery of drug targets and the development of new drugs. In our study, we also tested the prediction performance of WRMF on the most commonly used miRNA‐disease database (HMDD V2.0), which has 10,381 experimentally verified miRNA–disease associations. For HMDD V2.0, as shown in Figure 5A, WRMF obtains an AUC value of 0.9128 in five‐fold CV, in comparison to BNNR (0.8982), CMF (0.8899), and IMC (0.8363). As shown in Figure 5B, WRMF achieves an AUPR value of 0.4007, outperforming that of BNNR (0.3720), CMF (0.3807), and IMC (0.2507). Additionally, WRMF identified 656 associations in the top 1000 rankings, while BNNR, IMC, and CMF only predicted 634, 648, and 547 associations, respectively (see Figure 5C). Figure 5 indicates that WRMF performs better than the other comparison methods. The results on two different types of datasets prove that WRMF is generally a good model in association prediction.

### 3.5 Case study: WRMF identified the potential drugs for COVID‐19

COVID‐19 is a brand‐new (i.e. there is no interaction between COVID‐19 and any drug) and zoonotic disease. To further validate the prediction performance of WRMF, we conducted a case study to predict novel anti‐COVID‐19 drugs from a computational perspective. Specifically, we put the other known drug–virus associations to as the input of WRMF, then ranked the predicted scores of the potential anti‐COVID‐19 drugs. Following previous studies,^45^, ^55^ we adopted ClinicalTrials.gov and the Comparative Toxicogenomics Database (CTD)^56^ as references to validate whether the predicted drugs for COVID‐19 are efficacy or not. Table 1 shows that eight out of ten drugs (80% success rate) are validated by the reliable source, clinical trials, and previous literatures. For example, ribavirin (ranked the second) was predicted by WRMF to have an interaction with COVID‐19. Such a prediction can be supported by Clinicaltrials.gov and CTD. Nitazoxanide (ranked the fourth), a broad‐spectrum anti‐infective drug, can inhibit COVID‐19 at low micromolar concentrations (EC50 = 2.12 M).^57^ In addition, chloroquine (ranked the third), camostat (ranked the fifth), favipiravir (ranked the sixth), and remdesivir (ranked the eighth) predicted by WRMF have been confirmed by both CTD and clinical trials for COVID‐19 promising treatment. In summary, eight out of ten WRMF‐predicted anti‐COVID‐19 drugs were verified by the evidences from ClinicalTrials.gov and CTD. It indicates that WRMF offers a useful tool to prioritize potential repurposed drugs for COVID‐19.

**TABLE 1:** The Top 10 potential COVID‐19‐associated drugs predicted by WRMF on our constructed drug–virus dataset

DrugBank IDs	Candidate drugs	Evidences
DB00756	Hexachlorophene	NA
DB00811	Ribavirin	ClinicalTrials.gov, CTD, PMID:32222463
DB00608	Chloroquine	ClinicalTrials.gov,CTD, PMIDs:32070753, 32,173,110
DB00507	Nitazoxanide	ClinicalTrials.gov, PMID:33361100
DB13729	Camostat	ClinicalTrials.gov, CTD, PMID:33676899
DB12466	Favipiravir	ClinicalTrials.gov, CTD, PMID:32346491
DB15660	N4‐Hydroxycytidine	NA
DB14761	Remdesivir	ClinicalTrials.gov, CTD, PMIDs:32145386, 32,445,440
DB00218	Moxifloxacin	ClinicalTrials.gov, PMID:32546446
DB06803	Niclosamide	ClinicalTrials.gov, PMID:34664162

**FIGURE 6:**
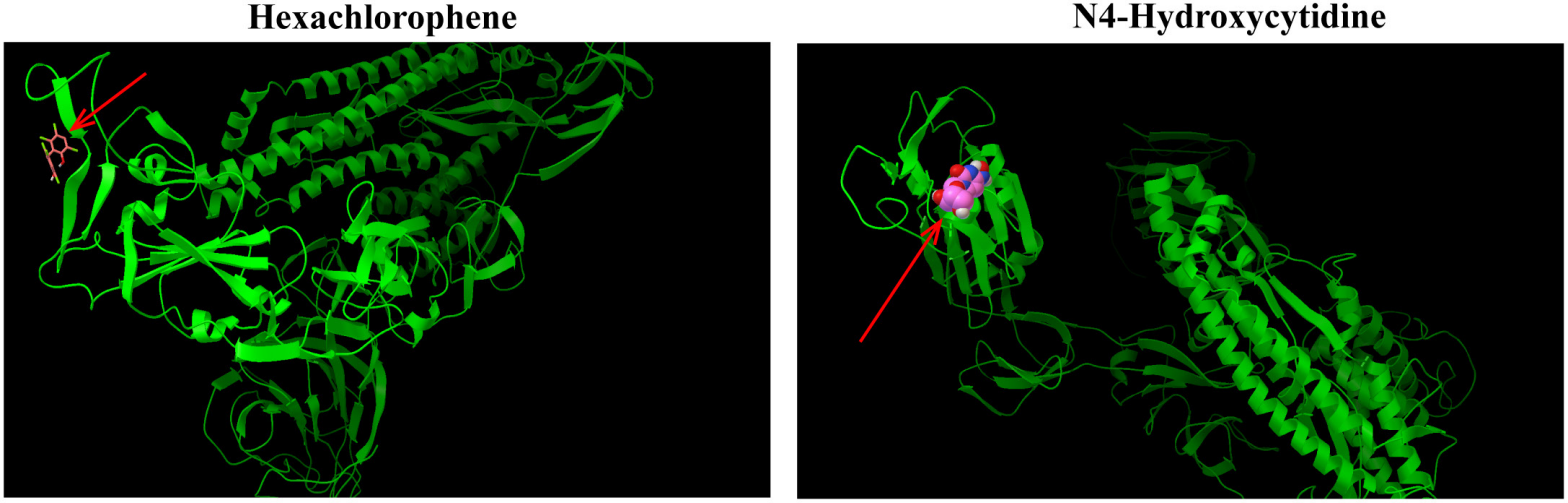
The predicted ligand‐protein binding mode between the two unconfirmed potential anti‐COVID‐19 drugs and the receptor ACE2 (angiotensin conversion Enzyme 2) using molecular docking

Second, molecular docking research is a method that provides valuable information and can be used to design well‐known ligands for specific active sites of large molecules. This is an economic and modern trend in drug discovery, where the technology‐based ligand–protein interaction reveals the possibility of pre‐synthesis. Hexachlorophene (ranked the first) and N4‐Hydroxycytidine (ranked the seventh) were conducted blind docking both in online and offline modes. The Autodock 4.2 package (http://autodock.scripps.edu) was used for offline docking. The X‐ray crystal structures of protein were retrieved from the RCSB protein database (www.rscb.org). A macromolecule with PDB ID: 6LZG, which is a novel coronavirus spike receptor binding domain complexed with its receptor ACE2. All proteins and ligands were prepared using MGL Tools 1.5.6 and Autodock Tool (ADT). ADT is used to calculate the binding free energy and inhibition constant of the optimal docking complex of the aforementioned proteins. The negative value of the combined free energy further indicates the stability of the complex (Table 2). Additionally, Figure 6 reveals that the two unproven drugs predicted by WRMF interact with multiple residues on its receptor ACE2 and once again shows that the drugs discovered by WRMF may have an inhibitory effect on COVID‐19.

**TABLE 2:** The binding affinity of top 10 drugs predicted by WRMF to the Target PDB ID: 6LZG

Rank	Candidate drugs	Free Energy of Binding (kcal/mol)
1	Hexachlorophene	−5.4
2	Ribavirin	−7.6
3	Chloroquine	−6.4
4	Nitazoxanide	−6.6
5	Camostat	−7.8
6	Favipiravir	−5.5
7	N4‐Hydroxycytidine	−5.6
8	Remdesivir	−6.8
9	Moxifloxacin	−6.5
10	Niclosamide	−8.1

**FIGURE 7:**
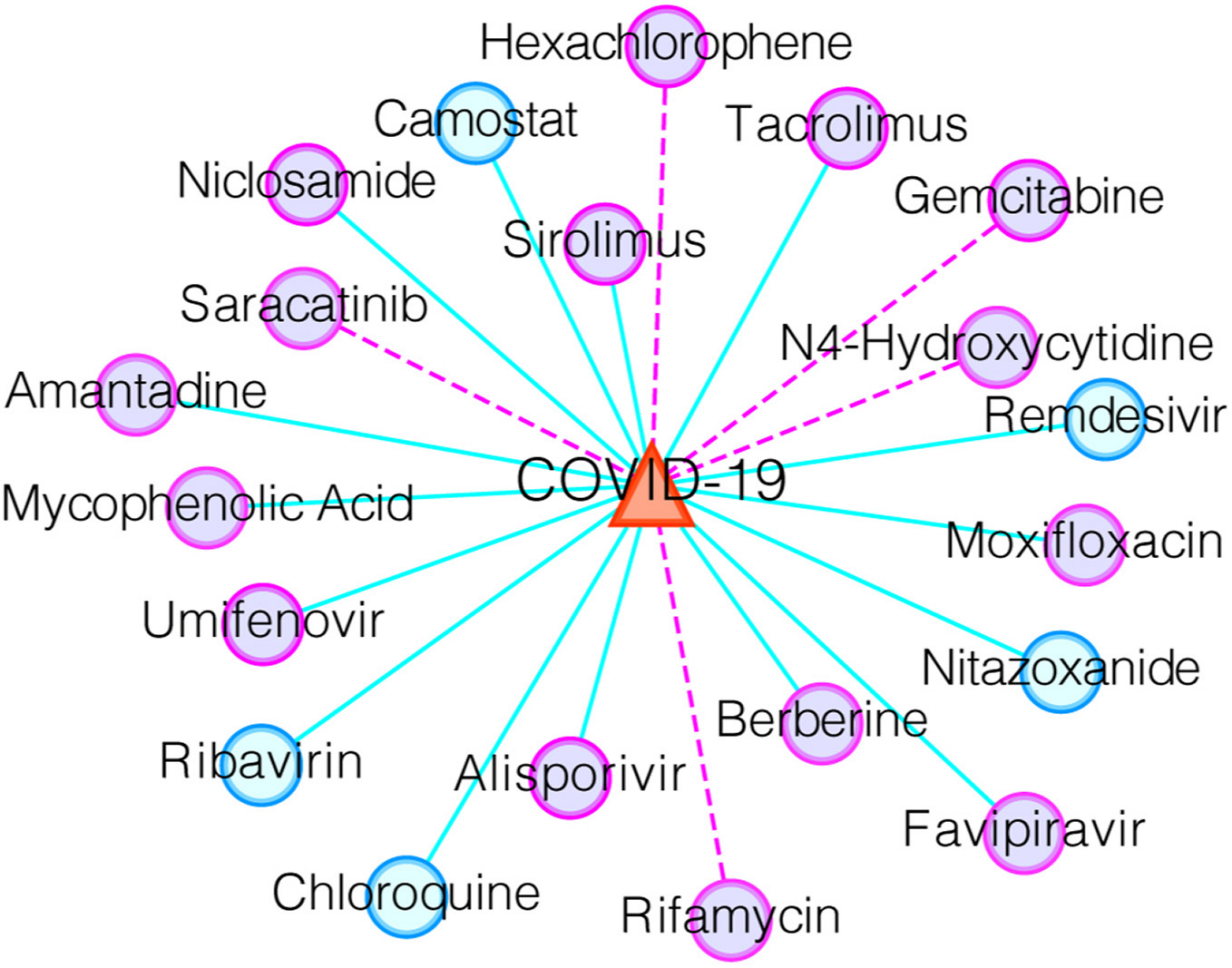
Top 20 anti‐COVID‐19 drug candidates predicted by WRMF on our constructed drug–virus dataset

Next, we listed the top 20 WRMF‐predicted anti‐COVID‐19 drugs based on our constructed drug–virus dataset (see Figure 7 and Table S1). As shown in Figure 7, some verified drug–virus associations are shown as light blue lines, while potential relationships are shown as magenta lines.

Finally, as the number of people infected with SARS‐CoV‐2 continues to increase, the drug–virus database is also increasing. In order to assess the expansibility and practicality ability of WRMF, we also applied WRMF to the DVA dataset^38^ from the study of Mongia et al. by comparing it with different types of approaches, including network‐based prediction method: MBiRW,^23^ deep learning‐based method: NIMCGCN,^25^ and several matrix factorization‐based algorithms: GRMF,^58^ GRMC,^59^ and WGRMF.^58^ The top‐10 predicted anti‐COVID‐19 drugs by these algorithms have been listed in Table 3. We validated the top‐10 candidate drugs of these algorithms by Clinicaltrials.gov. The bold font in Table 3 indicates that the predicted candidate drug has been validated by ClinicalTrials.gov. As can be seen, our proposed method WRMF obtains seven ClinicalTrials.gov‐validated drugs, more than that of GRMF, GRMC, and WGRMF. The promising clinical results signify that the practicality of BGNN in predicting potentially drugs for COVID‐19.

**TABLE 3:** The top‐10 anti‐COVID‐19 drugs predicted by WRMF and the other five algorithms based on the DVA dataset

MBiRW	NIMCGCN	GRMF	GRMC	WGRMF	WRMF
**Remdesivir**	**Remdesivir**	**Remdesivir**	**Remdesivir**	**Remdesivir**	**Umifenovir**
**Ribavirin**	**Ribavirin**	**Ribavirin**	**Umifenovir**	**Umifenovir**	**Ribavirin**
Taribavirin	Taribavirin	**Umifenovir**	**Ribavirin**	**Ribavirin**	**Remdesivir**
**Sofosbuvir**	Boceprevir	Taribavirin	Taribavirin	**Sofosbuvir**	Taribavirin
Vidarabine	Vidarabine	**Sofosbuvir**	**Sofosbuvir**	Paritaprevir	**Ibuprofen**
**Umifenovir**	Tecovirimat	Baloxavir marboxil	Vidarabine	Taribavirin	**Sofosbuvir**
Ganciclovir	Ganciclovir	Geldanamycin	Tenofovir alafenamide	Boceprevir	**Chloroquine**
Foscarnet	Foscarnet	Tenofovir alafenamide	Nelfinavir	Tenofovir alafenamide	Baloxavir marboxil
Cidofovir	Amprenavir	Tecovirimat	Amprenavir	**Favipiravir**	Peramivir
Didanosine	Didanosine	Peramivir	Boceprevir	**Chloroquine**	**Favipiravir**

*Note:* The bold font indicates that the candidate drug has been validated by ClinicalTrials.gov.

## 4 DISCUSSION

In this study, we proposed a novel in silico drug repositioning approach for uncovering the potential associations between viruses and drugs, termed WRMF. Apart from the known drug–virus association network via literature mining, we integrated one drug–drug chemical structure similarity network, and one virus–virus genome sequencing similarity network to construct a heterogeneous network, which contains a comprehensive view for screening anti‐COVID‐19 drug candidates. We have validated the prediction ability of WRMF in terms of five‐fold CV, the local LOOCV, two additional datasets validation, and a case study. The results show that our method achieves state‐of‐the‐art performance for repurposing anti‐COVID‐19 drugs. In future studies, since WRMF is a scalable approach, collecting and incorporating more relevant association data from more databases and literatures might improve its power.

We acknowledged several potential limitations in the current study. Although we take sizeable efforts to collect experimentally reported drug–virus associations from published literature, data quality is unassured and the drug–virus association data may be incomplete. We provided the top 20 WRMF‐predicted anti‐COVID‐19 drugs. State‐of‐the‐art pharmaco‐epidemiologic analysis on patient data (e.g. health insurance claims data) and in vitro or in in vivo mechanistic studies for the WRMF‐predicted anti‐COVID‐19 candidates are required in the future.

In summary, our findings suggest that in silico drug repurposing could benefit from constraints on drug and viral similarity, matrix factorization, and drug–virus heterogeneous network. WRMF could help offer novel efficacious therapies for multiple complex diseases if broadly applied.

## AUTHOR CONTRIBUTIONS

**Junlin Xu:** Conceptualization (equal); data curation (equal); methodology (equal); project administration (equal); writing – original draft (equal). **Yajie Meng:** Conceptualization (equal); data curation (equal); visualization (equal); writing – review and editing (equal). **Lihong Peng:** Conceptualization (equal); project administration (equal). **Lijun Cai:** Conceptualization (equal); supervision (equal). **Xianfang Tang:** Data curation (equal). **Yuebin Liang:** Visualization (equal). **Geng Tian:** Writing – review and editing (equal). **Jialiang Yang:** Conceptualization (equal); supervision (equal).

## CONFLICT OF INTEREST

The authors confirm that there are no conflicts of interest.

## Supporting information


Figures S1‐S2

Table S1

## Data Availability

The source code and data of WRMF is located at https://github.com/JunlinXu/WRMF
