# Supplementary material for: Computational drug repositioning using similarity constrained weight regularization matrix factorization: A case of COVID‐19
Source: J Cell Mol Med. 2022 May 29;26(13):3772–82. doi: 10.1111/jcmm.17412 (PMC9258716; doi:10.1111/jcmm.17412)
Supplement: Supplementary file 1 — Figures S1‐S2 [file JCMM-26-3772-s002.docx]

# Supplementary materials

## Title: Computational drug repositioning using similarity constrained weight regularization matrix factorization: a case of COVID-19

**Figures**


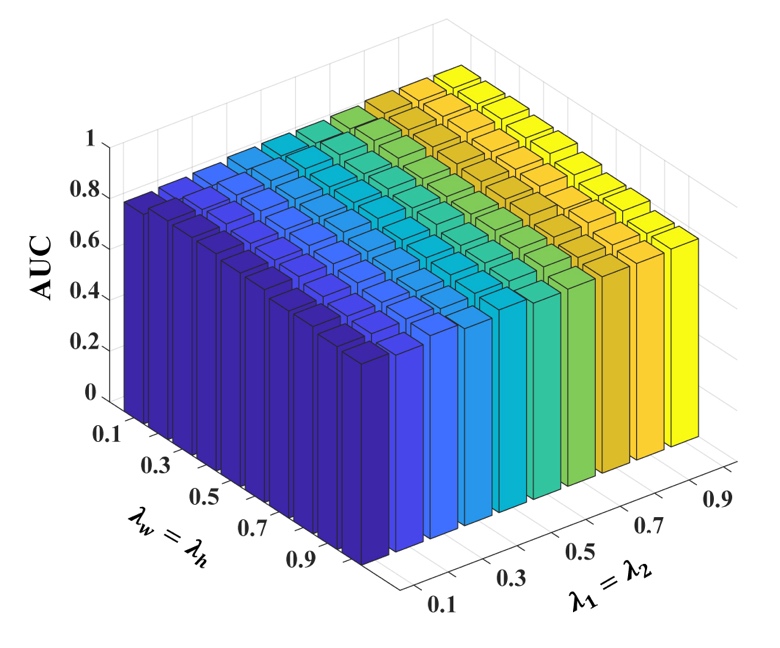


**Supplementary Figure 1.** The influence of main parameters on the performance of our proposed method WRMF.


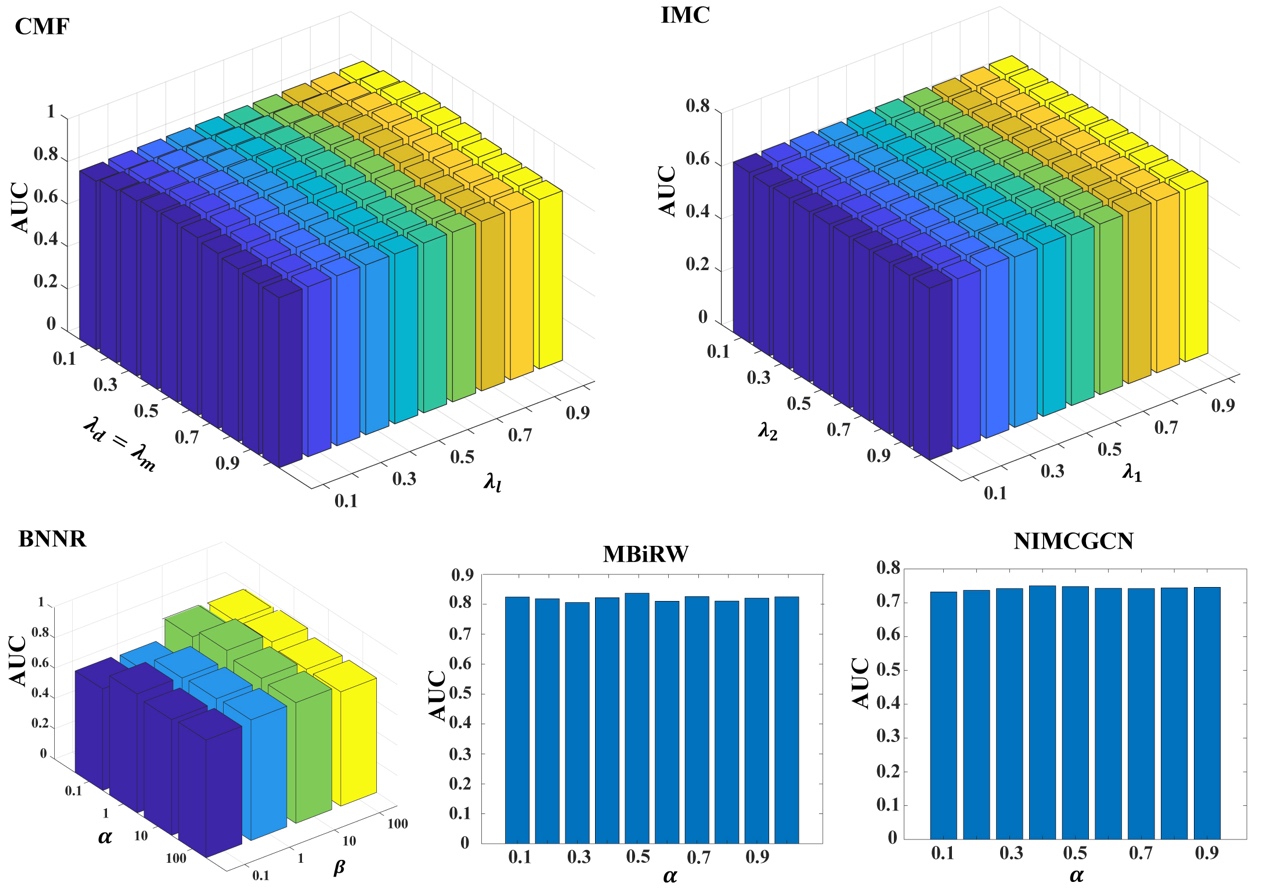


**Supplementary Figure 2.** The influence of main parameters on the performance of other comparison approaches.
