# Supplementary material for: Computational drug repositioning using similarity constrained weight regularization matrix factorization: A case of COVID‐19
Source: J Cell Mol Med. 2022 May 29;26(13):3772–82. doi: 10.1111/jcmm.17412 (PMC9258716; doi:10.1111/jcmm.17412)
Supplement: Supplementary file 2 — Table S1 [file JCMM-26-3772-s001.docx]

**Supplementary materials**

**Title: Computational drug repositioning using similarity constrained weight regularization matrix factorization: a case of COVID-19**

**Table**

**Supplementary Table 1.** Top 20 potential drugs predicted by WRMF that may treat COVID-19

| Rank | DrugBank IDs | Candidate drugs | Evidences |
| --- | --- | --- | --- |
| 1 | DB00756 | Hexachlorophene | Unconfirmed |
| 2 | DB00811 | Ribavirin | ClinicalTrials.gov,CTD, |
| 3 | DB00608 | Chloroquine | ClinicalTrials.gov,CTD |
| 4 | DB00507 | Nitazoxanide | ClinicalTrials.gov |
| 5 | DB13729 | Camostat | ClinicalTrials.gov,CTD |
| 6 | DB12466 | Favipiravir | ClinicalTrials.gov,CTD |
| 7 | DB15660 | N4-Hydroxycytidine | Unconfirmed |
| 8 | DB14761 | Remdesivir | ClinicalTrials.gov,CTD |
| 9 | DB00218 | Moxifloxacin | ClinicalTrials.gov |
| 10 | DB06803 | Niclosamide | ClinicalTrials.gov |
| 11 | DB13609 | Umifenovir | ClinicalTrials.gov,CTD |
| 12 | DB00915 | Amantadine | ClinicalTrials.gov |
| 13 | DB01024 | Mycophenolic Acid | ClinicalTrials.gov |
| 14 | DB11753 | Rifamycin | Unconfirmed |
| 15 | DB00864 | Tacrolimus | ClinicalTrials.gov |
| 16 | DB12139 | Alisporivir | ClinicalTrials.gov |
| 17 | DB00877 | Sirolimus | ClinicalTrials.gov,CTD |
| 18 | DB04115 | Berberine | ClinicalTrials.gov |
| 19 | DB00441 | Gemcitabine | Unconfirmed |
| 20 | DB11805 | Saracatinib | Unconfirmed |
